# A Retrospective Evaluation of the Retrosigmoidal Approach for Petroclival Meningioma Surgery and Prognostic Factors Affecting Clinical Outcome

**DOI:** 10.3389/fonc.2022.786909

**Published:** 2022-04-01

**Authors:** Waseem Masalha, Dieter Henrik Heiland, Christine Steiert, Marie T. Krueger, Daniel Schnell, Christian Scheiwe, Anca-L. Grosu, Oliver Schnell, Juergen Beck, Juergen Grauvogel

**Affiliations:** ^1^ Department of Neurosurgery, Medical Center—University of Freiburg, Freiburg, Germany; ^2^ Faculty of Medicine, University of Freiburg, Freiburg, Germany; ^3^ Department of Neurosurgery, Cantonal Hospital St. Gallen, St. Gallen, Switzerland; ^4^ Department of Radiation Oncology, Medical Center—University of Freiburg, Freiburg, Germany; ^5^ German Cancer Consortium (DKTK), Partner Site Freiburg, Freiburg, Germany

**Keywords:** meningioma, surgery, postoperative radiotherapy, petroclival meningioma, progression-free survival

## Abstract

**Introduction:**

Petroclival meningioma (PCM) remains a major neurosurgical challenge. There are still controversial strategic treatment concepts about surgical approach, the extent of resection, and postoperative radiotherapy. We aimed to evaluate prognostic factors influencing the progression-free survival (PFS) rates of PCM, with a particular focus on the retrosigmoidal approach, the role of the extent of resection, and postoperative radiotherapy.

**Methods:**

Eighty-nine patients with complete follow-up data were included. All patients were operated on *via* a retrosigmoidal approach, of whom 19 underwent gross total resection (GTR) and 70 underwent subtotal resection (STR). In the subgroups of tumors with infiltration of the cavernous sinus, 41 patients received near total resection (NTR) and 24 STR. Thirty-one patients received postoperative radiotherapy of the residual tumor and 58 were treated with surgery alone. Kaplan–Meier analyses and Cox regression were used to identify significant factors associated with treatment.

**Results:**

GTR (*p*=0.0107) and postoperative radiotherapy (*p*=0.014) were associated with significantly improved PFS. Even the subgroup analysis of extended PCM with infiltration of the cavernous sinus (CS) showed an advantage for PFS after near total resection (NTR) (*p*=0.0017). The additional radiotherapy of the residual tumor in the CS in this subgroup also showed a beneficial effect on PFS (*p*=0.012).

**Conclusion:**

The extension of surgical resection remains the most important prognostic factor in relation to oncological outcomes. However, the GTR of extended PCM with infiltration of the CS is associated with significant neurological morbidity and requires additional adjuvant therapy concepts. Postoperative radiotherapy is an important element in the treatment of the residual tumor after surgery.

## Introduction

Meningiomas are usually benign lesions that account for a total of 20%–25% of intracranial tumors. About 10% of meningiomas occur in the posterior fossa, of which 5%–11% affect the petroclival region (1, 2). As defined by Al-Mefty et al. (3), true petroclival meningiomas (PCMs) are lesions arising from the upper two-thirds of the clivus with the dural attachment centered on the petroclival junction. They are located medial to the internal auditory meatus and posterior to the gasserian ganglion. PCM can extend into the cavernous and petrosal sinus, middle cranial fossa, parasellar region, tentorium, foramen magnum, Meckel’s cave, and various cranial nerve foramina before they manifest clinically (4, 5).

Surgical resection of petroclival meningioma remains challenging due to their deep location and attachment to vital neurovascular structures. In the past, the resection of petroclival meningioma was associated with a high rate of morbidity and mortality (1, 5–7). The introduction of precise skull base techniques and advances in microsurgery have significantly improved the clinical outcome, leading to less mortality and morbidity (8–12).

Several complex skull base approaches have been developed to resect petroclival meningioma and to provide wider access to the tumors such as the subtemporal transpetrosal and extended middle fossa approaches, transcochlear or translabyrinthine, and combined infra- and supra-tentorial approaches (9, 13–15). However, these approaches require extensive drilling of the temporal bone, a complex and time-consuming procedure with a high risk of morbidity and complications such as hearing loss, injury to the facial nerves, or temporal lobe vascular injury (16). The retrosigmoidal approach was described as a less-invasive approach with a low rate of postoperative complications; however, this approach was only applied to certain tumors (9, 10, 13). We have been tending to apply the retrosigmoidal approach as a less-invasive approach, in all tumors, as a treatment strategy to provide the best chance of complete removal of the tumors while minimizing the potential for postoperative complications.

As radiosurgery has become more advanced, many authors recommend subtotal resection of the PCM and subsequent radiotherapy of residual tumors, thus minimizing surgical morbidity and improving quality of life (17–19). On the other hand, radiosurgery can prevent tumor progression, but it is limited to small- to medium-sized tumors (20, 21). In addition, the risk of new or worsening symptoms is increased in petroclival-localized processes after radiosurgery (22).

The aim of this retrospective study was to investigate the retrosigmoidal approach and extent of surgical resection, the influence of additional postoperative radiotherapy after surgery on progression-free survival in patients with true petroclival meningioma and to evaluate prognostic factors that affect the outcome and the clinical course of petroclival meningioma.

## Materials and Methods

The present study is a retrospective, single-center study that included patients with PCM who were operated on in our Department of Neurosurgery between 1998 and 2018.

Patients were included in the study only if they fulfilled the following criteria: (1) aged older than 18 years, (2) histopathological diagnosis of meningioma WHO I at the time of surgery, and (3) patients with true petroclival meningioma. The local ethics committee of the University of Freiburg, Germany, approved the study. Informed consent was obtained from all patients.

### Data Acquisition

Patient gender, age at the time of surgery, primary/secondary tumors, presence of edema and compression of the brainstem based on preoperative MRI, involvement of the cavernous sinus, tumor size, extent of resection, and recurrence/progression were collected. Only true PCMs as defined by Al-Mefty et al. were included (3).

The extent of surgical resection was assessed by surgical reports and 3-month follow-up MR imaging according to the Simpson grading scale (18). The gross total resection was defined as Simpson grades I and II and the incomplete resection or subtotal resection as Simpson grades III–V. However, the gross total resection of tumors with cavernous sinus involvement using a retrosigmoidal approach is not achievable. Therefore, these tumors were evaluated in the subgroup analysis as follows: near-total resection of posterior fossa was defined when the surgical report and postoperative MRI showed only a residual tumor in the cavernous sinus area. The subtotal resection was defined if a residual tumor was present in the region of the cavernous sinus and in the posterior fossa.

**Figure 1:**
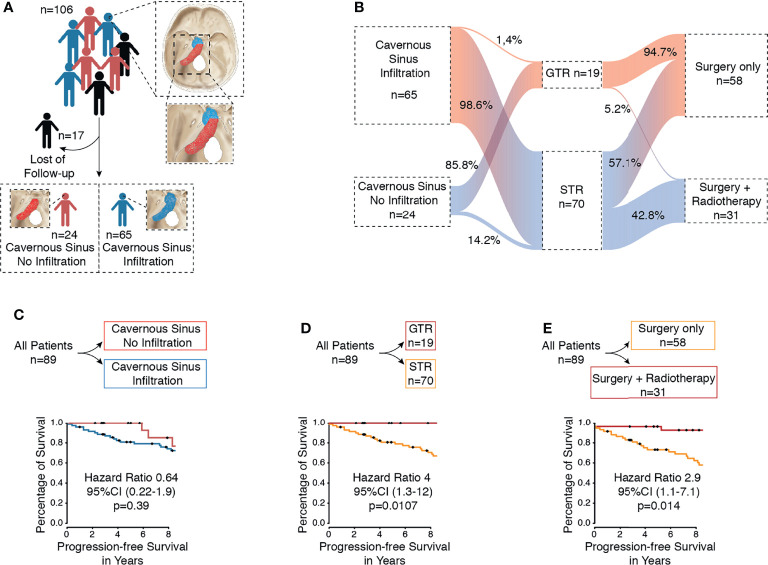
**(A)** Flow diagram of included patients with petroclival meningioma from our database. **(B)** Flow diagram of the different treatment arms. **(C)** Kaplan–Meier curve of all patients for progression-free survival based on infiltration of cavernous sinus. **(D)** Kaplan–Meier curve of all patients for progression-free survival based on extent of resection (GTR vs. STR). **(E)** Kaplan–Meier curve of all patients for progression-free survival based on therapy (surgery vs. surgery plus radiotherapy).

The mean follow-up was 11 ± 6.9 years (range, 0.9–27.9 years). All patients underwent frequent MRI scans 3 months postoperatively and at a regular interval of 1 year. The Karnofsky Performance Scale (KPS) and progression-free survival (PFS) were used to assess oncological/neurological outcomes. New lesions or growing residual tumors on a follow-up MRI scan were defined as tumor progression. Two independent investigators assessed MRI scans. Patients with incomplete record data were excluded (**Figure 1A**). Tumors of the lower third of the clivus are by definition foramen magnum tumors and were therefore excluded (8). Furthermore, we excluded lateral petrous and petrotentorial lesions included in other series because tumors at these sites present a much lower risk of cranial nerve injury during surgery (5, 11).

### Surgical Approach and Resection Grade

All patients included in this study were operated in our neurosurgical department using a retrosigmoidal approach in the semi-sitting position under neuromonitoring supervision of the cranial nerves and transesophageal echocardiography to detect air embolisms. After positioning the patient and fixing the head with a clamp, a retromastoid skin incision was performed, followed by exposure of the suboccipital bone and a retrosigmoid craniectomy. Under microscopic view, the dura was opened in a U-shape, taking into consideration the border to the transverse and sigmoid sinuses. Subsequently, the cerebellomedullary cistern was opened for the drainage of cerebrospinal fluid (CSF). The cerebellum was slightly retracted to expose the tumor. The tumor was resected from its attachment and debulked as far as possible. Parts of the petrous bone were drilled to gain wider access, depending on the extent of the tumor extension. Following tumor removal, successful hemostasis was ensured, and the dura was closed watertight. The lateral margin of the craniectomy was also closed with a muscle patch to prevent postoperative rhinoliquorrhea due to potentially opened mastoid air cells. Simpson grading was assessed by surgical reports. To minimize bias between surgeons, Simpson grading was reassessed by postoperative MRI at 3 months.

### Tumor Size

The tumor size was measured from the preoperative MRI scan and the radiologist’s report. The largest diameter in the anterior–posterior, transverse, or craniocaudal dimension was used as a general measurement of tumor size.

### Radiation Regime

Fractionated high-precision radiotherapy was performed in 31 patients. Treatment planning was based on CT and MRI according to institutional guidelines. Fractionated treatment was prescribed with a median dose of 54 Gy in single fractions of 1.8 Gy. Follow-up included a clinical examination and contrast-enhanced imaging. All patients were followed up prospectively after radiotherapy in our radiotherapy department as part of a rigorous follow-up regimen.

### Tumor Histology

According to standard procedures, tissue samples were fixed with a 4% phosphate-buffered formaldehyde and embedded in paraffin. Using standard protocols, H&E staining was applied to 4-μm paraffin sections. Immunohistochemistry was conducted with an autostainer (Dako) after heat-induced epitope retrieval in citrate buffer.

### Statistical Analysis: Cox Regression

In this study, the primary endpoint was PFS. PFS was defined as the time interval between surgery and tumor recurrence/progression diagnosed on the follow-up MRI scans, as recently described (23). First, Cox regression was performed in a univariate manner. Significant parameters were further tested by multivariate analysis as recently described in detail (23). We defined the alpha-level as 5% without adjustment to reach a statistical power at a minimum of 80%. All statistical analyses were performed using an R-software tool (package: survival, ggplot2, MANOVA) and IBM SPSS statistics version 22.

### Data Visualization

Plots were performed by an R-software package ggplot2 and *tidyverse*.

### Statistical Analysis: Group Comparison

To determine significance in differences between our analyzed parameters, we considered significance at an alpha level below 5% (p < 0.05). The following parameters were taken into consideration: age, sex, Simpson grade, preoperative and postoperative KPS, and tumor size. Distribution and variances of all data were tested by a Shapiro–Wilk test (*p*>0.05) to confirm normality. We tested the difference between both groups by a Wilcoxon signed-rank test (unpaired) for numeric variables a chi-square test or Fisher’s exact test for nominal variable and determined a 5% alpha-level. Test statistics were performed as recently described (23).

## Results

### Patient Data

Between 1998 and 2018, a total of 106 patients with true petroclival meningioma were treated in the Department of Neurosurgery. A total of 17 patients were excluded due to a lack of follow-up data (**Figure 1A**). The sex ratio (male/female) was 1:5.84. First, patients were divided based on the treatment (**Table 1**). The surgery group included 58 patients (8 male and 50 female) with a median age of 56.5 years (confidence interval 95%, 39.7–73.2), and the surgery plus postoperative radiotherapy group included 31 patients (5 male and 26 female) with a median age of 54 years (CI 95%, 42–68.5). In the following subgroup analysis, patients were divided based on tumor infiltration of the cavernous sinus. We identified 24 (27%) patients with PCM without an infiltration of the cavernous sinus and 65 (73%) patients with PCM with an infiltration of the cavernous sinus (**Figures 1A, B**). Frequent symptoms at presentation were headache, gait disturbance, dizziness, hydrocephalus, and cranial nerves deficits. A detailed overview of all parameters is given in **Table 4**.

**Table 1:** Patient data.

Parameter	Surgery	Surgery plus Radiotherapy	
	N=58	N=31	
Age (median, CI 95%)	56.5 (39.7–73.2)	54 (42–68.5)	*p*=0.088*
Sex (N, %)			*p*=0.76**
** Female**	50 (86%)	26 (84%)	
**Male**	8 (14%)	5 (16%)	
Resection grade (N, %)			p=0.0054***
**GTR**	18 (31%)	1 (3%)	
**STR**	40 (69%)	30 (97%)	
Preoperative KPS (median, CI 95%)	80 (70–90)	80 (70–90)	*p*=0.94*
Postoperative KPS (median, CI 95%)	90 (80–100)	80 (70–100)	*p*=0.44*
Edema on Brainstem	19 (32%)	10 (32%)	*p*=1***
No edema on brainstem	39 (68%)	21 (68%)	*p*=1***
Tumor size in cm³ (median, CI 95%)	10 (4.7–24.7)	15 (14–39)	*p*=0.32*
Primary	50 (86%)	24 (77%)	*p*=0.44***
Secondary	8 (14%)	7 (23%)	*p*=0.44***
Compression of brainstem	51 (88%)	29 (94%)	*p*=0.48**
No compression of brainstem	7 (12%)	2 (6%)	*p*=0.48**

^*^Wilcoxon test.

^**^Fisher,s exact test.

^***^Chi-squared test.

KPS, Karnofsky Performance scale; CI, confidence interval; GTR, gross total resection; STR, subtotal resection.

### Tumor Extension

First, we aimed to give an overview of the tumor extension and its effect on the PFS. A total of 65 (73%) patients with petroclival meningioma showed infiltration of the cavernous sinus and 24 (27%) patients without infiltration of the cavernous sinus (**Figure 1A**). We investigated whether the infiltration of the cavernous sinus affected the PFS. The Kaplan–Meier analysis showed no difference between both groups (*p*=0.39) (**Figure 1C**).

### Extent of Tumor Resection According to Simpson Grade of All Patients

All patients underwent surgical resection of the tumor. In 19 cases (21.3%), the gross total resection (Simpson grades I and II) was achieved, and 70 cases (78.7%) received a subtotal resection (Simpson grades III, IV, and V) (**Figures 1B, D**). A tumor recurrence was observed in one patient after a gross total resection (Simpson grades I and II). In contrast, 28 patients (31.4%) showed progression after a subtotal resection (Simpson grades III, IV, and V). The difference between both groups was statistically significant (*p*=0.0107) (**Figure 1D**). The difference was also significant in univariate analysis (*p*= 0.011) and multivariate analysis (*p*=0.005) (**Table 2**).

**Table 2:** Cox-regression analysis of all patients.

Variable clinical and treatment factors	Progression-free survival		Progression-free survival	
	HR (95% CI)	*p-*value	HR (95% CI)	*p*-value
	Univariate analysis		Multivariate analysis	
**Sex (female vs. male)**	0.42 (0.18–0.99)	*0.048*	0.33 (0.12–0.92)	*0.034*
**Age (≥55 vs. <55)**	0.34 (0.15–0.78)	*0.011*	0.28 (0.11–0.69)	*0.005*
**GTR vs. STR**	4 (1.3–12)	*0.015*	4.5 (1.04–19.4)	*0.005*
**Surgery vs. surgery plus radiotherapy**	2.9 (1.1–7.1)	*0.024*	7 (2.42–20.3)	*0.0003*
**Primary vs. Secondary**	0.84 (0.34–2.1)	0.7		
**Preoperative KPS (≥80 vs. <80)**	0.31(0.12–0.76)	*0.011*	0.52 (0.17–1.5)	0.25
**Postoperative KPS (≥80 vs. <80)**	0.34 (0.14–0.79)	*0.012*	0,58 (0.2–1.6)	0.3
**Presence of edema on the brainstem**	0.46 (0.22–0.97)	*0.041*	0.6 (0.26–1.3)	0.22
**Involvement of cavernous sinus**	0.64 (0.22–1.9)	0.42		
**Compression of brainstem**	1.8 (0.61–5.2)	0.29		
**Tumor size (≥19 cm³ vs. <19 cm³)**	1.7 (0.79–3.7)	0.17		

GTR, gross total resection; STR, subtotal resection; CI, confidence interval; HR, hazard ratio; KPS, Karnofsky Performance scale.fi 2.

### Extent of Tumor Resection in Patients Without Infiltration of Cavernous Sinus

**Figure 2:**
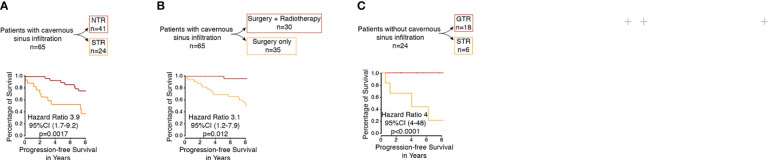
**(A)** Kaplan–Meier curve of patients with PCM with infiltration of the cavernous sinus for progression-free survival based on extent of resection (NTR vs. STR). **(B)** Kaplan–Meier curve of patients with PCM with infiltration of the cavernous sinus for progression-free survival based on therapy (surgery vs. surgery plus radiotherapy). **(C)** Kaplan–Meier curve of patients PCM without infiltration of the cavernous sinus for progression-free survival based on extent of resection (GTR vs. STR).

Within the subgroup analysis, 18 patients with petroclival meningioma without an infiltration of the cavernous sinus received a GTR and six patients received an STR. Tumor recurrence was observed in one patient (4%) after a gross total resection (Simpson grades I and II). On the contrary, five patients (20.8%) showed progression after subtotal resection (Simpson grades III, IV, and V). The difference between both groups was statistically significant (*p*=0.0001) (**Figure 2C**). Uni- and multivariate analyses in this subgroup were not reasonable due to the small number of patients.

### Extent of Tumor Resection in Patients With Infiltration of the Cavernous Sinus

Gross total resection of PCM with an infiltration of the cavernous sinus is often associated with severe morbidity. Therefore, in our own clinic, the operation aimed for maximal safe resection, paying special attention to the decompression of the posterior fossa. Near total resection (NTR) was defined when the tumor was completely resected in the region of the posterior fossa and a residual tumor was left in the cavernous sinus. Subtotal resection was defined when the residual tumor was left in the cavernous sinus and posterior fossa.

In this series, a total of 65 patients presented with PCM with infiltration of the cavernous sinus, of which 40 patients (62%) received an NTR, one patient received a GTR (1%), and 24 (37%) patients received an STR. Tumor recurrence was observed in nine patients (13%) after an NTR. Thirteen patients (20%), however, showed progression in the region of the posterior fossa after an STR. The difference between both groups was statistically significant (*p*=0.0017) (**Figure 2A**). The difference was also significant in the univariate analysis (*p*=0.0018) and the multivariate analysis (*p*=0.0008) (**Table 3**).

**Table 3:** Cox-regression analysis of patient with infiltration of cavernous sinus.

Variable clinical and treatment factors	Progression-free survival		Progression-free survival	
	HR (95% CI)	*p*-value	HR (95% CI)	*p*-value
	Univariate analysis		Multivariate analysis	
**Sex (female vs. male)**	0.39 (0.16–0.97)	*0.044*	0.41 (0.15–1.08)	0.07
**Age (≥55 vs. <55)**	0.54 (0.23–1.3)	0.17		
**STR vs. NTR**	3.9 (1.7–9.2)	*0.0018*	5.2 (1.9–13.7)	*0.0008*
**Surgery vs. surgery plus radiotherapy**	3.1(1.2–7.9)	*0.02*	4.8 (1.6–14.3)	*0.004*
**Primary vs. secondary**	1.7 (0.49–5.7)	0.41		
**Preoperative KPS (≥80 vs. <80)**	0.39 (0.16–0.98)	*0.045*	1.5 (0.44–5)	0.52
**Postoperative KPS (≥80 vs. <80)**	0.37 (0.15–0.94)	*0.036*	0.53 (0.16–1.7)	0.3
**Presence of edema on the brainstem**	0.69 (0.29–1.6)	0.4		
**Compression of brainstem**	2.4 (0.69–8.2)	0.17		
**Tumor size (≥19 cm³ vs. <19 cm³)**	1.3 (0.57–3.1)	0.5		

GTR, gross total resection; NTR, near total resection; CI, confidence interval; HR, hazard ratio.

### Postoperative Radiotherapy of All Patients

Fractionated stereotactic radiotherapy was performed when a residual tumor was seen in the postoperative 3-month MRI. Thirty-one patients (34.8%) were treated postoperatively with stereotactic radiotherapy of the remaining tumor, of which six patients showed tumor recurrence/progression (6.7%). All tumors were treated with a radiation dose between 54 and 57 Gy. In contrast, 58 patients (65.1%) were treated with surgery alone, of which 21 patients (23.5%) showed recurrence/progression of the tumor. The Kaplan–Meier analysis (*p*=0.014) (**Figure 1E**) and the univariate (*p*=0.024) and multivariate analyses (*p*=0.0003) showed significant differences between both groups (**Table 2**).

### Postoperative Radiotherapy in Patients With Infiltration of the Cavernous Sinus

In the performed subgroup analysis of tumors with infiltration of the cavernous sinus, 30 patients (46.1%) were treated with postoperative stereotactic radiotherapy of the residual tumor in the region of the cavernous sinus of which six patients (9.2%) showed tumor recurrence/progress. In contrast, 35 patients (53.8%) were treated with surgery alone without postoperative radiotherapy, of which 16 (24.6%) showed a tumor recurrence/progress. The statistical analysis showed significant differences between both groups (*p*=0.012) (**Figure 2B**). The additional univariate analysis (*p*=0.02) and multivariate analysis (*p*=0.004) showed also significant differences between both groups (**Table 3**).

### Postoperative Radiotherapy in Patients Without Infiltration of the Cavernous Sinus

In the subgroup of patients with PCM without infiltration of the cavernous sinus, only one patient was postoperatively irradiated. This patient did not show any tumor recurrence.

### Surgical Outcome

The most affected cranial nerves after surgery were the oculomotor nerve (n=10, 11.2%), the facial nerve (n=17, 19.1%), and the vestibulocochlear nerve (n=17, 19.1%), of which six patients (6.7%) had a permanent oculomotor nerve deficit, nine patients (10.1%) had a permanent facial nerve deficit, and six (6.7%) had a permanent vestibulocochlear nerve deficit. A detailed overview of all deficits of the cranial nerves is given in **Table 4**.

**Table 4:** Postoperative cranial nerve deficits of patients who underwent surgery.

Postoperative cranial nerve deficits	Early (N, %)	Permanent (N, %)
II c.n	1 (1.1%)	1 (1.1%)
III c.n	10 (11.2%)	6 (6.7%)
IV c.n	4 (4.4%)	1 (1.1%)
V c.n	10 (11.2%)	5 (5.6%)
VI c.n	15 (16.8%)	7 (7.8%)
VII c.n	17 (19.1%)	9 (10.1%)
VIII c.n	17 (19.1%)	6 (6.7%)
IX c.n	2 (2.2%)	0 (0%)
X c.n	2 (2.2%)	1 (1.1%)
XI c.n	2 (2.2%)	2 (2.2%)
XII c.n	1 (1.1%)	1 (1.1%)

c.n, ranial nerve.

Other postoperative surgical morbidities occurred in 17 patients (19%), including hydrocephalus (n=11), tracheostomy (n=1), motor weakness (n=1), intracranial hematoma (n=2), consciousness disorder (n=1), intracranial infection (n=1), and cerebrospinal fluid leak (n=2). Three patients received a GTR, and 14 received an STR. The mean preoperative KPS was 80% ± 9% (range, 60%–100%), and the mean postoperative KPS was 80% ± 11% (range, 50%–100%).

### Other Factors Influencing PFS of All Patients

Additional univariate and multivariate Cox regression analyses of patients with progressive disease was performed to identify potential prognostic factors for tumor recurrence/progression. The univariate analysis (*p*=0.011) and the multivariate analysis (*p*=0.005) of all patients showed a potential for improved PFS in patients under 55 years of age compared to patients over 55 years of age (**Table 2**). Sex was also included in this analysis; the univariate analysis (*p*=0.048) and the multivariate analysis (*p*=0.034) of all patients showed that men have better PFS (**Table 2**). With regard to the presence of edema on the brain stem, a significant difference was found in the univariate analysis (*p*=0.041). In the multivariate analysis, however, no difference was found (*p*=0.22) (**Table 2**). No significance was detected with regard to the compression of brainstem and tumor size.

### Other Factors Influencing PFS in Patients With Infiltration of the Cavernous Sinus

Univariate and multivariate analyses of the subgroup PCM with infiltration of the cavernous sinus showed no significant factors prolonging PFS, except for the extent of resection and postoperative radiotherapy (**Table 3**).

### Tumor Histopathology

All operated PCMs in this series were WHO grade I meningioma. We identified 78 (87.6%) meningothelial, five (5.6%) transitional, four (4.4%) psammomatous, and two (2.2%) angiomatous meningioma.

## Discussion

This study retrospectively reviewed patients with true petroclival meningioma operated on between 1998 and 2018 and is one of the largest single-institutional series published in the literature regarding petroclival meningioma. The aim of this retrospective study was to investigate the retrosigmoidal approach, the extent of surgical resection, and the influence of additional postoperative radiotherapy after surgery on progression-free survival in patients with true petroclival meningioma and to determine prognostic factors that affect the outcome and the clinical course of petroclival meningioma.

### Surgical Approach

All patients in our institution were operated by retrosigmoidal approach in a semi-sitting position. The retrosigmoidal approach allows access to the petrosal surface of the temporal bone. Extensive skull base approaches can significantly increase surgical morbidity and are associated with postoperative neurological deficits (24). Therefore, the simple retrosigmoidal approach has gained more and more interest (24).

The sitting position was first introduced in 1913 by De Martel and modified (semi-sitting position) by Madjid Samii (25). The advantages of a semi-sitting position are lowered cerebral venous pressure and intracranial pressure during the surgery. In our cohort, the semi-sitting position was the preference of the surgeons in our clinic due to the reasons abovementioned. This position promotes gravity drainage of blood and irrigation fluid, thus keeping the surgical field clear at all times. The disadvantage of placing the patient in a semi-sitting position include a risk of tension pneumocephalus, venous air embolism, and the increased fatigue of the hands of a surgeon. The semi-sitting position is controversially discussed in the literature. Some papers show no increased risk associated with this position (26, 27), while others show an increased risk associated with this position compared to other neurosurgical positions (28, 29). Our experience has shown that a semi-sitting position is feasible with acceptable risk even in patients with patent foramen ovale (PFO). This is in line with other reports (30–32). Although the combined petrosal approach offers a better presentation of the surgical site, this approach is associated with increased morbidity, postoperative CSF leakage, prolonged operative time, and increased risk of damage to the facial nerve. The subtemporal approach is easier, but the vena of Labbé usually limits the elevation of the temporal lobe. The risk of sensory aphasia due to damage of the left temporal lobe increases (15, 33).

### Surgery: Extent of Resection

The extent of resection remains the most important factor for outcome in patients with benign meningioma. Since the Simpson grading is based on subjective intraoperative observation, we also defined the extent of resection based on pre- and postoperative MRI. GTR was defined as Simpson grades I and II, i.e., when no macroscopic tumor was left intraoperatively and no enhancing regions were present on the postoperative imaging. STR was defined as Simpson grades III–IV. NTR was defined when the tumor was completely resected in the region of the posterior fossa and the residual was left in the cavernous sinus.

By these criteria, 21.3% (19 of 89) of all patients had GTR, and 78.7% (70 of 89) had STR. Other major series reported GTR rates of 20%–85% (5, 14, 19, 34, 35). However, these series may include different subtypes of petroclival region tumors other than true petroclival meningioma as investigated here. We found that the GTR of petroclival meningioma was associated with significantly better PFS (*p*=0.0107) (**Figure 1D**). The additional multivariate analysis also showed significantly better PFS (*p*=0.005, **Table 2**). In the subgroup analysis, this was also the case for PCM without cavernous sinus infiltration (*p*=0.0001, **Figure 2C**).

In a PCM with infiltration of the cavernous sinus, a GTR by definition cannot be achieved without high morbidity. Nevertheless, a significantly better PFS after GTR of the posterior fossa, i.e., after NTR (*p*=0.0017, **Figure 2A**) (*p*=0.0008, **Table 3**), was found. These results emphasize the importance of the degree of resection, even in patients with infiltration of the cavernous sinus. These results are in line with other major published studies (3, 36, 37). Al-Mefty et al. also highlighted the importance of GTR and reported that the cavernous sinus extension had no negative effect on the extent of resection in their series. Therefore, they recommended performing a complete resection despite the infiltration of the cavernous sinus (3). Many others recommend a restrictive surgical strategy to minimize neurological deficits to maintain a high quality of life (5, 19, 38–40). Couldwell et al. reported a tumor recurrence in 14 patients after an STR in their series, of which 12 residual tumors were located in the cavernous sinus (5). However, the resection of the cavernous tumor portion is associated with an increased risk of neurological deficits (36). We did not strategically aim to resect the tumor portion in the cavernous sinus to minimize the neurological deficits. In this analysis, we found no significant difference regarding PFS in tumors with and without infiltration of the cavernous sinus (*p*=0.39, **Figure 1C**).

### Radiotherapy

The main challenge in the therapy of petroclival meningioma is the treatment of large tumors in which a complete resection is often not possible because critical neighboring structures such as the cavernous sinus, cranial nerves, or large vessels are also involved. Subtotal resection is usually performed when there is an invasion of the cavernous sinus. Before 1970, stereotactic radiotherapy and radiosurgery were not considered to be effective in the treatment of meningioma until early studies showed a reduced rate of local recurrence after postoperative radiotherapy (41).

We found better PFS favoring all patients submitted to postoperative radiotherapy in the Kaplan–Meier analysis (*p*=0.014) (**Figure 1E**), and the additional multivariate analysis also demonstrated significantly better PFS (*p*=0.0003) (**Table 2**). We also found in the subgroup analysis of PCM with infiltration of the cavernous sinus that the postoperative radiotherapy was associated with significantly better PFS in the Kaplan–Meier analysis (*p*=0.012) (**Figure 2B**). The additional multivariate analysis also demonstrated significantly better PFS (*p*=0.004) (**Table 3**). These results are in line with those of other reports (13, 21, 42, 43). Sekhar and Schramm first recommended postoperative radiotherapy for partially resected petroclival meningioma in 1987 (44). Feng Xu et al. recommended radiosurgery for petroclival meningiomas under consideration of patient age, size, location of the residual tumor, and pathological characteristics (13). Flannery et al. even suggested that radiosurgery should be considered as a first-line treatment for patients with small symptomatic petroclival meningioma (45). Others recommended postoperative radiotherapy only in the case of a recurrent tumor or regrowth of the residual tumor detected by MRI (14, 46). Others have shown that stereotactic radiotherapy is an effective and safe treatment for the local control of cavernous sinus meningioma with a low risk of significantly late toxicity, especially cranial nerve deficits (42, 43, 47, 48). These results are consistent with our findings (**Figure 2B**).

However, the controversy remains. Al-Mefty et al. highlighted in their series that GTR (grade I or II) of petroclival meningioma was possible in 76.4% of cases including tumors with infiltration of the cavernous sinus. The authors suggested that if circumstances prevent GTR, residual tumors could be managed by watchful waiting until progression, at which time a new intervention could be planned (3). Other groups also emphasized the importance of radical resection (46). Moreover, Starke et al. reported that clival- or petrous-based locations indicate an increased risk of a new or worsening neurological deficit after stereotactic radiotherapy (22).

### Cranial Nerves Deficits

The largest series reported that postoperative CN deficits ranged from 20.3% to 67% (14, 49). In our series, we observed 33% permanent CN deficits (**Table 4**). However, the CNs affected postoperatively are not necessarily the ones that were affected preoperatively. Al-Mefty and colleagues demonstrated that CNs V and VIII were most likely to improve following surgery, while CN VI was most likely to be permanently injured (3). Similar results were reported by Natarajan and colleagues (14). Furthermore, other studies report that CN VII and V were the most postoperatively injured (40, 49). In our studies, CN III, V, VI, VII, and VIII were the most frequently affected cranial nerves at an early stage. The CN III, V, and VII have recovered worst later on (**Table 4**). The postoperative KPS was included in our study as an assessment parameter of the surgical outcome. Multivariate statistical analysis conducted in respect of PFS demonstrated no significant differences between patients with a postoperative KPS below 80 and those with a postoperative KPS above 80 (**Table 2**). Similar results were also reported by other groups (36, 42).

### Predictors of Progression-Free Survival

Risk predictors for PFS of PCMs have been demonstrated in several studies (5, 19, 36). In our series, age (<55 years) was a prognostic factor for a better outcome (**Table 2**). These results are in line with those of other reports (14, 17). In our study, the difference between genders could be confirmed as an independent predictive factor in the univariate and multivariate analyses (**Table 2**). In contrast, other studies reported no difference between both groups (36). The presence of edema on the brainstem, tumor size, and compression of the brain stem were not predictors of PFS in the present study. In contrast, others reported that the presence of edema and the compression of the brain stem might affect the degree of resection (19, 50).

### Limitations of the Study

This study is limited by its retrospective and observational nature, which may have led to selection bias, and by the external validity within a single institution. Additional limitations imposed by a retrospective study design, such as heterogeneous management strategies without random assignment, variability in the extent of follow-up, and variability between observers in assessing the extent of resection, must be considered when interpreting the results. Another limitation of this study is the number of patients lost to follow-up (n=17).

Due to the retrospective nature of the study, postoperative radiotherapy was not randomly assigned; instead, it was recommended according to the skull base tumor board assessment, which is another risk of bias.

Nonetheless, our study is one of the largest series to date, focusing on the extent of resection of true petroclival meningioma and their postoperative radiotherapy.

## Conclusions

Petroclival meningioma remains a surgical challenge. The retrosigmoid approach has the advantages of less invasiveness and a shorter operation time. The most important prognostic factor in determining recurrence was the extent of resection according to Simpson grading. However, radical resection is frequently associated with various neurological deficits due to the infiltration of the cavernous sinus and other neurovascular structures. In this study, the additional postoperative radiotherapy significantly increased the progression-free survival of the residual tumor in the region of the cavernous sinus after near complete resection, although the use of postoperative radiotherapy remains controversial in the management of petroclival meningioma. Prospective randomized trials should be performed to define the role of radiotherapy in the management of patients with petroclival meningioma, beyond the conflicting evidence from the existing retrospective series.

## Data Availability Statement

The raw data supporting the conclusions of this article will be made available by the corresponding author, without reservation.

## Ethics Statement

The studies involving human participants were reviewed and approved by the local ethics committee oft he University of Freiburg, Germany. The patients/participants provided their written informed consent to participate in this study.

## Author Contributions

WM drafted the manuscript and participated in the data collection. DHH participated in the preparation of the manuscript and in the data collection. WM, DHH, and MTK participated in the design of the figures. OS, JB, DS, CSc, and ALG participated in the preparation of this article by revising it with regard to important intellectual content. CSt and JG coordinated the study and revised the article for important intellectual content. All authors contributed to the article and approved the submitted version.

## Funding

The article processing charge was funded by the Baden-Wuerttemberg Ministry of Science, Research and Art and the University of Freiburg in the funding programme Open Access Publishing.

## Conflict of Interest

The authors declare that the research was conducted in the absence of any commercial or financial relationships that could be construed as a potential conflict of interest.

## Publisher’s Note

All claims expressed in this article are solely those of the authors and do not necessarily represent those of their affiliated organizations, or those of the publisher, the editors and the reviewers. Any product that may be evaluated in this article, or claim that may be made by its manufacturer, is not guaranteed or endorsed by the publisher.
